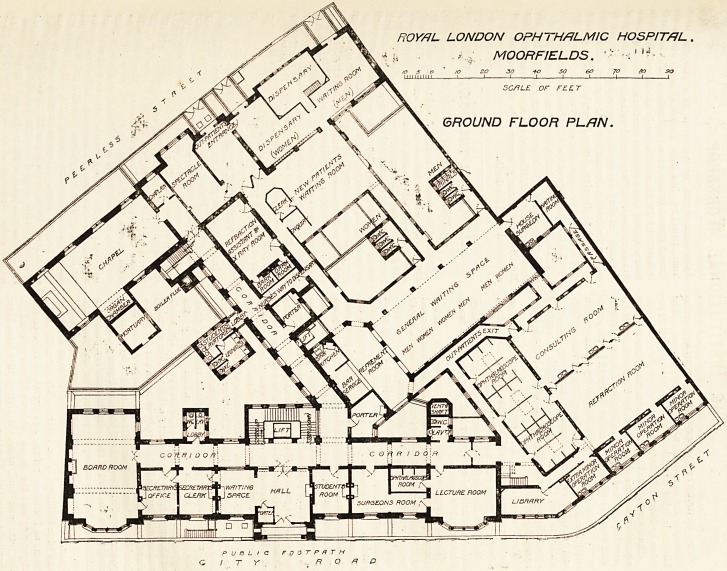# Hospital Construction

**Published:** 1900-07-28

**Authors:** 


### The Institutional Workshop.

### HOSPITAL CONSTRUCTION.

### THE ROYAL LONDON OPHTHALMIC HOSPITAL, CITY ROAD, E.C.

The site on which the new hospital has been built is an irregular-shaped piece of land some 35,000 ft. in area, having frontages to City Road, Cayton Street, and Peerless Street. So far as the ground floor is concerned, the ground is to a very large extent covered with buildings; but on the level of the first floor the plan consists of three distinct blocks, viz., the front block, facing City Road; the back or Peerless Street block, and an intermediate block parallel with the last, with the connecting-link between the two. The greater part of the ground floor is taken up by the out-patient department, which it will be convenient to describe by itself.

#### Out-patient Department.

The entrance for out-patients is in Peerless Street. Passing through the entrance lobby patients will find themselves opposite to the glass enclosure forming the clerk's office. Here all new patients will be registered, and will receive their letters. Immediately facing the porter's office is a waiting-room for new patients, capable of seating some 200 persons. Adjoining the clerk's office is an office for the official whose duty it is to make inquiries in certain cases into the circumstances of intending recipients of the charity. From here the patients pass down a corridor to a large waiting-hall, where old and new patients are divided into three groups corresponding to the three surgeons on duty for the day. From here they pass by relays into the consulting-room, a large hall 45 ft. by 24 ft., with accommodation for three surgeons and three assistants. The light for this room is obtained from a large skylight in the adjoining room, the partition between the two rooms being almost entirely of glass. Communicating with this room is a separate room for the out-patient surgical officer with a small waiting space attached. Next the consulting-room is the refraction-room (45 ft. by 29 ft.), and opening out of this is a series of four small rooms for minor operations. At the side of and communicating with both consulting and refraction rooms is a large dark room for ophthalmoscope work with compartments for 18 patients. The exit passage for patients is placed at the other side of the dark room, and is connected also directly both with the refraction-room and the consulting-room, so that patients not needing to be examined in the dark room or not wanted in the refraction-room may be sent away direct from either room. On the line of route of outgoing patients is a refreshment bar with a small kitchen. Beyond this is a room for *x*-ray work with dark-room attached, and close to the dispensary and exit door is the spectacle-room. Patients can either leave direct from the spectacle-room, or if they have to go to the dispensary can do so by passing through a turnstile, and so to the waiting-room for medicine. This waiting-room is divided into two, with the dispensary placed in the middle. In the latter are three serving windows —one for in-patients, the other two for out-patients.

In the basement, underneath the whole space occupied by dispensary and waiting-rooms, is a store for drugs and bottles, &c. Separate groups of water-closets are provided for the use of out-patients in two courtyards connected with the waiting-room by covered ways. In planning the out-patient department care has been taken to prevent, as far as possible, the possibility of patients meeting or crossing one another, and also to render the passage from one part to another as direct as possible. The whole of the walls in this department, with the exception of the ophthalmoscope-room, are lined with glazed bricks, and the floors throughout with terrazzo.

In the matter of ventilation and warming the out-patients' department is treated entirely separately from the other parts of the hospital. Fresh air, forced in by a large fan worked by a water motor and passed through an air filter provided with a water spray, is conveyed by trunks to the various rooms. Provision for warming the air in cold weather is made by means of batteries of hot water pipes placed in the air trunks. For cooling the air in summer, if found necessary, there is ample means of introducing ice into the air trunks at suitable points. The vitiated air is extracted by a separate set of trunks and discharged at the top of a shaft above the roof of the main buildings. At the foot of the shaft is a fan for use as an auxiliary to the inlet fan when required.

A staircase placed at the junction of the main front block with the Cayton Street block leads to the museum and pathological laboratories. These rooms are immediately over the minor operation-rooms and the library. The pathological department comprises a general laboratory, with accommodation for six workers, a dark-room for photographic work, and a private laboratory for the curator. The staircase is carried up to the second floor to afford ready access for the curator to the operation theatre. The whole of the basement floor and the remainder of the ground floor is devoted to administrative offices. In the basement are stores of all kinds, the boiler-house, disinfecting-room, and porters'-room.

On the ground floor in the centre of the City Road block is the main entrance hall, with porter's lodge and waiting space; to the left are the secretary's offices and board-room, and to the right a room for students, one for the honorary medical staff, and a lecture-room. Separate lavatory accommodation is provided for: (*a*) medical officers; (*b*) secretary; and (*c*) students. On this floor are two porter's bed-rooms, and in the Peerless Street block the entrance for in-patients, visitors, and the chapel.

The first floor of the City Road block contains a bed-room and sitting-room each for two house surgeons, the staff dining-room with pantry adjoining, the matron's office, her sitting-room, bed-room, and bath-room.

The Peerless Street block contains two wards for 15 beds each, with a large day-room; the intermediate block contains a ward for 18 beds, one for two beds, a sister's room, bath-room, and linen-room; and in the connecting building are the following: Patients' lavatory, ward kitchen, two-bed ward, and two bath-rooms. Approached from the corridor connecting the three blocks by a covered bridge is the sanitary tower containing water-closets and sink-rooms.

On the second floor the arrangement of wards is similar to that on the first floor, except that a smoking-room takes the place of the two-bed ward in the connecting building.

The main front block has in the centre the operation theatre, with waiting and anæsthetic rooms on one side, and sterilising and recovery rooms on the other side. At the extreme right are two wards, one for four, the other for two beds; and on the left are two rooms for sisters and the nurses' common room. This room is provided with fitted furniture by Messrs. Hampton, designed specially by the architects.

The third floor contains in the front the kitchen, offices, servants' hall, nurses' dining-room, and seven bedrooms for servants, with two bath-rooms. The kitchen is fitted up with steam and gas cooking apparatus by Messrs. Slater and Co.

The intermediate block is on this floor, divided into separate rooms for sixteen nurses. These rooms are all provided with hot-water ventilating radiators, and fitted with specially planned fitments designed by the architects. Ventilation is provided for by an extraction shaft, formed by putting a false ceiling to the corridor, and provided with an extracting fan propelled by a water motor. In the connecting block is another nurses' bedroom, making 17 in all, and three bathrooms.

The Peerless Street block contains two wards, as on the floor below, one of which will be devoted to children, a large day-room, and a sisters' room.

A fourth floor is formed over the connecting building, and contains two wards for sick nurses, with a ward kitchen and bath-room.

The construction of the building throughout is what is usually known as fireproof, *i.e.*, the floors are formed of iron girders protected by terra-cotta tubes (Fawcett's patent system), and covered with coke breeze concrete, and all the partition walls are either of brick or of hollow terra-cotta tiles filled with concrete (Shepwood's patent). The floors of the corridors throughout are finished either with terrazzo or with artificial stone, and the staircases are made of cast concrete with iron joists embedded therein. The ward floors are laid with teak boards, side nailed direct to the concrete. The fireplaces throughout are provided with grates of the now well-known Teale pattern, the invention of Mr. T. Pridgem Teale, the distinguished surgeon. The walls and ceiling of the operation-room are lined with "opalite" and opaque tinted glass tile bedded in Keene's cement, the key for which is formed by fragments of glass fused on to the back of the tile. The floor is of terrazzo. The corridors and passages, bath-rooms, and lobbies to w.c.'s are warmed by hot-water radiators from the same boilers as those which supply the apparatus in the out-patients' department. The whole of the engineering work in connection with the warming and ventilating, and the supply of hot water for baths, sinks, &c., has been carried out by Messrs. Ashwell and Nesbitt. There are four lifts all worked by hydraulic power supplied from the Hydraulic Power Company's mains. In the well of the main staircase is a passenger lift which, besides conveying patients to and from the operation-room, will be used for the conveyance of clean linen. Off the connecting corridor, between the front and back buildings, is an external lift for coals, soiled linen, and kitchen refuse, and for the service of the dispensary and the stores entrance lifts are placed in the front and back areas. All these lifts have been supplied by the Otis Elevator Company, and are worked by hydraulic power. The building is lighted throughout by electric current from the Supply Company's mains, the work having been carried out by Messrs. Belshaw and Co., under the direction of Mr. Walton, C.E.

As already stated there are two frontages to the hospital—one towards City Road, the other Peerless Street. The City Road front being the main entrance, and being in a conspicuous part of a wide and important thoroughfare, it was thought necessary that some elaboration should be given to its appearance; this front, therefore, has been designed in a somewhat French type of Renaissance architecture. The building is faced with Portland stone up to the sills of the first floor windows, above which it is red brick with Portland stone dressings. A large and handsome porch occupies the centre of the building, in the carved panels of which appear the date of foundation and date of rebuilding. The front to Peerless Street is carried out in a very simple manner to harmonise with the plain, not to say squalid, surroundings of the neighbourhood.

The buildings were designed and carried out throughout by the architects, Messrs. Young and Hall, of 17, Southampton Street, Bloomsbury, with whom the late Mr. Bedells, formerly surveyor to the hospital, was associated until his death.

## Figures and Tables

**Figure f1:**